# Comparative analysis of myocardial strain parameters in acute decompensated heart failure phenotypes: a cross-sectional study

**DOI:** 10.3389/fcvm.2026.1794242

**Published:** 2026-07-09

**Authors:** Zhizhou Lin, Songyuan Yu, Jiajie Yin, Hong Jiang, Mengru Liu, Xianlun Li

**Affiliations:** 1 Department of Integrative Medicine Cardiology, China-Japan Friendship Hospital, Beijing, China; 2 China-Japan Friendship Hospital (Institute of Clinical Medical Sciences), Chinese Academy of Medical Sciences & Peking Union Medical College, Beijing, China

**Keywords:** acute decompensated heart failure, correlation, left ventricular ejection fraction, myocardial strain parameters, two-dimensional speckle tracking echocardiography

## Abstract

**Objective:**

To investigate the differences in two-dimensional speckle tracking echocardiography (2D-STE) parameters among patients with different types of acute decompensated heart failure (ADHF) and their correlation with left ventricular ejection fraction (LVEF).

**Methods:**

Ninety-eight patients hospitalized due to ADHF were included in this study. Patients were divided into two subgroups based on their LVEF using a cutoff of ≤49%: heart failure with reduced ejection fraction (HFrEF) and heart failure with preserved ejection fraction (HFpEF). All patients underwent transthoracic echocardiography and 2D-STE.

**Results:**

Compared to the HFrEF group,the HFpEF group showed significantly higher absolute values of GLSA4C, GLSA2C, GLSA3C, and LV-GLS values (*P* < 0.05), as well as higher absolute values of LASR-ED, LASRD-ED, LASRT-ED, RVFWSL, and RV4CSL values (*P* < 0.05). The HFrEF subgroup had higher LAFI and LASI values compared to the HFpEF group (*P* < 0.05). In the HFrEF subgroup, LVEF was negatively correlated with GLSA4C, GLSA2C, GLSA3C, LV-GLS, RVFWSL, RV4CSL, LASr-ED, LASrct-ED, as well as LAFI and LASI (*P* < 0.05). However, LVEF was positively correlated with LASrcd-ED (*P* < 0.05). In the HFpEF subgroup, LVEF was negatively correlated only with GLSA4C, GLSA2C, GLSA3C and LV-GLS (*P* < 0.05). After controlling for related variables (LVEF), the correlations between LV-GLS and LASr-ED, LASrcd-ED, and LASrct-ED remained significant in both groups of heart failure patients (*P* < 0.05). In HFrEF patients, the correlations between RVFWSL and LASr-ED, LASrcd-ED, and LASrct-ED disappeared (*P* > 0.05). In HFpEF patients, RVFWSL was negatively correlated with LASr-ED (*P* < 0.05), positively correlated with LASrct-ED (*P* < 0.05), but its correlation with LASrcd-ED disappeared (*P* > 0.05).

**Conclusion:**

Significant differences exist in 2D-STE parameters between the HFrEF and HFpEF groups, and LVEF correlates with these parameters. After adjusting for LVEF, LV-GLS remains correlated with various parameters in different heart failure groups. Our findings suggest a stronger interdependence between right ventricular and left atrial mechanics in HFpEF patients compared to HFrEF patients, evidenced by the persistence of correlations between RV free wall strain and LA reservoir/pump strain after adjusting for LVEF.

## 1 Introduction

Acute Decompensated heart failure (ADHF) is characterized by symptoms and signs such as dyspnea, exercise intolerance, palpitations, pre-syncope, peripheral edema, abdominal distension, early satiety, and fatigue (1, 2). The pathophysiological mechanisms underlying ADHF remain incompletely understood, leading to limited treatment options. Echocardiography is the preferred imaging modality for diagnosing cardiovascular diseases. Among its parameters, ejection fraction (EF) is widely used to assess cardiac systolic function. With advancements in echocardiography, novel imaging techniques and parameters have been developed to evaluate cardiac function. Although left ventricular ejection fraction (LVEF) plays a critical role in diagnosing heart failure, prognostication, patient classification, and guiding therapeutic decisions (3), it has certain limitations. For instance, LVEF may not fully reflect actual cardiac function and fails to differentiate between healthy and pathological states (4, 5).

Two-dimensional speckle tracking echocardiography (2D-STE) tracks the spatial motion of myocardial speckles, providing real-time insights into myocardial motion and deformation. 2D-STE strain parameters are simpler to obtain and exhibit high sensitivity, reproducibility, and are less dependent on loading conditions and ventricular geometry compared to LVEF (6). Strain analysis, as a novel technique, offers a comprehensive evaluation of myocardial contractility and deformation, helping to better characterize patient conditions (7). Numerous studies have identified global (endocardial) longitudinal strain of the left ventricle (LV-GLS) as a sensitive marker of myocardial dysfunction (3, 8). LV-GLS can aid in predicting the risk of future events and stratifying risk in heart failure patients (9). Its measurements are stable and highly reproducible, making it superior to LVEF in evaluating subclinical cardiac dysfunction and prognosis (10–12). LV-GLS is useful in assessing the risk of heart failure in patients with conditions such as diabetes, hypertension, cardiomyopathy, and coronary artery disease (13–16). Additionally, it can be employed for stratifying patients with heart failure with preserved ejection fraction (HFpEF) and for evaluating their prognosis and treatment outcomes during follow-up (17, 18).

Given the limited data on 2D-STE in ADHF patients, this study aims to investigate the differences in 2D-STE parameters among ADHF subtypes and examine the correlations between LVEF and 2D-STE parameters.

## 2 Methods

### 2.1 Study subjects

The study subjects met the following diagnostic criteria: cardiac and vascular dysfunction due to hemodynamic decompensation caused by various underlying conditions and triggers, presenting with clinical manifestations such as dyspnea, exercise intolerance, palpitations, pre-syncope, peripheral edema, abdominal distension, early satiety, and fatigue, defined as acute decompensated heart failure (ADHF) (19). Patients were initially stratified according to the 2021 ESC Guidelines into three groups: HFrEF (LVEF <40%), HFmrEF (LVEF 40%–49%), and HFpEF (LVEF ≥50%). However, the HFmrEF subgroup contained only 8 patients (8.2% of the total cohort), which was insufficient for robust statistical analysis. Therefore, based on the contemporary clinical overlap in guideline-directed medical therapy recommendations and the need for statistical power, the HFrEF and HFmrEF patients were combined into a single group (defined as LVEF <50%) for the primary analysis in this study. The original three-group comparison was explored but yielded no significant findings due to the limited sample size of the HFmrEF group. All participants were recruited under an approved ethical protocol (Ethics ID: 2024-ZF-32).

### 2.2 Research methodology

We prospectively enrolled patients hospitalized between September 2023 and July 2024 due to acute heart failure (AHF) or acute exacerbation of chronic heart failure. Based on medical records, these patients were confirmed to have congestive heart failure (CHF) and were discharged with a diagnosis of acute decompensated heart failure (ADHF). We recorded the patients' age, sex, height, weight, comorbidities, and cardiac functional classification. On the day of admission or the following day, we documented the following laboratory parameters:Total white blood cell count (WBC), total lymphocyte count (L), total neutrophil count, hemoglobin (Hb), platelets Creatine kinase (CK), serum uric acid (SUA), urea nitrogen (Urea), glucose (GLU), total cholesterol (TC), triglycerides (TG), high-density lipoprotein cholesterol (HDL-C), low-density lipoprotein cholesterol (LDL-C). Serum creatinine concentration (SCr), estimated glomerular filtration rate (eGFR). Cardiac markers: Myoglobin (Myo), troponin T (TnT), N-terminal pro-brain natriuretic peptide (NT-ProBNP), and creatine kinase isoenzyme (CK-MB).

### 2.3 Echocardiography examination

Transthoracic echocardiography was performed within 72 h of admission using a Phillips IE33 system by experienced cardiovascular ultrasound specialists. Patients were positioned in the left lateral decubitus position, and ECG gating was maintained during imaging. At the end of expiration, images from three cardiac cycles were captured for analysis. Parameters measured included LVEF%, early (E) and late (A) diastolic peak velocities at the mitral valve, early diastolic mitral annular velocity (e′'), and the E/e′ ratio. Additionally, LVEDD, LVESD, left atrial diameter (LAd), right atrial transverse diameter (RATD), and right ventricular basal diameter (RVD1) were recorded.

### 2.4 Measurement of LV strain and strain rate

We selected the apical four-chamber view (GLSA4C), apical two-chamber view (GLSA2C), and apical three-chamber view (GLSA3C). Using specialized software plugins, the system automatically delineated a multicolored, “horseshoe-shaped” region of interest (ROI) along the left ventricular myocardium.

Manual adjustments were made if automatic featuring failed to track myocardium properly. In the strain curves from the AP4 and AP2 planes, the peak value of left ventricular global longitudinal strain (LV-GLS) was identified. The average value of LV-GLS was measured and recorded.

### 2.5 Measurement of LA strain and strain rate

The AP4 and AP2 planes were selected for observation. From the strain rate curves in these planes, the following measurements were taken: Left atrial global average strain rate during systole (LASr), Left atrial global average strain rate during early diastole (LAScd), and Left atrial global average strain rate during late diastole (LASct). The average values for each strain rate parameter were calculated and recorded.

### 2.6 Measurement of RV strain and strain rate

Key parameters included RV global longitudinal strain (RVGLS) and RV free wall strain (RVFWS). RVGLS represented the average strain of the interventricular septum and RV free wall segments, while RVFWS included only the three RV free wall segments. RVFWS was preferred for assessing RV function due to reduced accuracy of RVGLS, which is influenced by LV contraction.

## 3 Statistical analysis

Data analysis was conducted using SPSS 27.0 statistical software. The methodology for statistical evaluation included the following: Continuous Variables: Data conforming to a normal distribution were expressed as mean ± standard deviation (±s). Group comparisons were performed using the independent sample *t*-test for two groups and one-way ANOVA for multiple groups. Data not following a normal distribution were described using median and interquartile range [M(P25, P75)]. Comparisons between groups utilized the independent sample Wilcoxon rank-sum test (Mann–Whitney *U* test) to ensure accuracy. Correlation Analysis:Pearson correlation analysis was employed for normally distributed variables. Spearman correlation analysis was used for variables not following a normal distribution. Multivariable linear regression analyses were further performed to evaluate the independent association between LV-GLS and LASr-ED in different HF phenotypes. A *P*-value <0.05 was considered statistically significant, indicating meaningful differences or correlations.

## 4 Results

### 4.1 Demographic and clinical characteristics of heart failure patients

A total of 98 cases were included in this study, with clinical characteristics summarized in Table 1. The average age of all patients was 64.28 ± 13.589 years, and the body mass index (BMI) was 24.49 (21.96–27.32) kg/m^2^. Among the patients, 80 (81.63%) were male. Comorbid conditions included:70 patients (71.4%) with coronary artery disease,61 patients (62.2%) with hypertension, 50 patients (51%) with hyperlipidemia, and 42 patients (42.9%) with diabetes mellitus.

**Table 1:** Demographic and clinical characteristics of heart failure patients.

Variable	Overall (*n* = 98)	HFrEF group (*n* = 46)	HFpEF group (*n* = 52)	*P*-value
Age (years)	64.28 ± 13.58	63.46 ± 13.02	65.27 ± 14.14	0.513
Systolic BP (mmHg)	129.99 ± 19.86	126.74 ± 18.55	132.92 ± 20.71	0.126
Diastolic BP (mmHg)	77.91 ± 14.12	78.63 ± 13.02	77.25 ± 15.14	0.634
Male, *n* (%)	80 (81.6)	38 (82.6)	42 (80.8)	0.814
Smoking, *n* (%)	43 (43.9)	18 (39. 1)	25 (48.1)	0.373
Alcohol use, *n* (%)	30 (30.6)	11 (23.9)	19 (36.5)	0.176
Diabetes, *n* (%)	42 (42.9)	21 (45.7)	21 (40.4)	0.599
CAD, *n* (%)	70 (71.4)	31 (67.4)	39 (75)	0.405
Previous MI, *n* (%)	33 (33.7)	18 (39. 1)	15 (28.8)	0.282
Hypertension, *n* (%)	61 (62.2)	29 (63)	32 (61.5)	0.878
Hyperlipidemia, *n* (%)	50 (51)	22 (47.8)	28 (53.8)	0.552
Hyperuricemia, *n* (%)	21 (21.4)	9 (19.6)	12 (23.1)	0.672
Atrial Fibrillation, *n* (%)	12 (12.2)	5 (10.9)	7 (13.5)	0.696
Stroke, *n* (%)	21 (21.4)	10 (21.7)	11 (21.2)	0.944
Anemia, *n* (%)	13 (13.3)	7 (15.2)	6 (11.5)	0.592
Arrhythmia, *n* (%)	11 (11.2)	5 (10.9)	6 (11.5)	0.917
CKD, *n* (%)	24 (24.5)	11 (23.9)	13 (25)	0.901
Cardiomyopathy, *n* (%)	7 (7. 1)	4 (8.7)	3 (5.8)	0.703
BMI (kg/m^2^)	24.49 (21.96–27.32)	24.45 (22.39–27.04)	24.63 (21.92–27.51)	0.991
HDL-C (mmol/L)	0.99 (0.84–1.15)	0.94 (0.81–1.09)	1.03 (0.85–1.30)	0.027
NT-proBNP (log10)	2.79 (2.16–3.50)	3.19 (2.28–3.57)	2.54 (2.11–3.29)	0.045
Use of Sacubitril/Valsartan	33 (33.7)	24 (52.2)	9 (17.3)	<0.001

There were 52 patients (53.06%) with HFpEF and 46 patients (46.94%) with HFrEF. Statistically significant differences (*P* < 0.05) were observed between the two groups in the following parameters:NT-ProBNP: Higher in HFrEF patients. HDL-C: Higher in HFpEF patients. Usage of sacubitril/valsartan (Entresto): More frequent in HFrEF patients.

### 4.2 Echocardiographic analysis

The results of the echocardiographic analysis revealed several significant differences between the HFrEF and HFpEF groups. Specifically, the HFpEF group had larger values for LVEDD, LVESD, RATD, RVD1, and E/e′, while the LAd and LVEF values were smaller compared to the HFrEF group (*P* < 0.05). There were also notable differences in the size of both ventricles and atria between the two groups. Details are presented in Table 2.

**Table 2:** Echocardiographic analysis.

Variable	Overall (*n* = 98)	HFrEF (*n* = 46)	HFpEF (*n* = 52)	*P*-value
LAd	40.29 ± 8.33	42.43 ± 7.78	38.40 ± 8.41	0.016
LVEDD	55 (50–61)	60 (55–65)	51.5 (46–55)	<0.01
LVESD	40 (33–48)	48 (42–54)	33 (30–38)	<0.01
RATD	36 (32–39.25)	36.72 (33–41)	35 (30–38)	0.031
RVD1	34 (29.75–38)	35 (31–40)	32.5 (28–35.75)	0.004
E/e′	11.3 (9–17.92)	15.55 (10.42–20.3)	10.1 (8.25–12.35)	<0.01
LVEF	50.5 (40.22–60.75)	39 (33.25–44.75)	59.5 (53–66.75)	<0.001

### 4.3 2D speckle tracking analysis

Compared to the HFrEF group, the HFpEF group exhibited significantly higher absolute values of values of GLSA4C, GLSA2C, GLSA3C, and LV-GLS (*P* < 0.05). Additionally, absolute values of LASR-ED, LASRD-ED, LASRT-ED, RVFWSL, and RV4CSL were elevated in the HFpEF group (*P* < 0.05). Conversely, the HFrEF subgroup demonstrated higher values of LAFI and LASI compared to the HFpEF group (*P* < 0.05). Details are presented in Table 3.

**Table 3:** 2D speckle tracking analysis.

Variable	Overall (*n* = 98)	HFrEF (*n* = 46)	HFpEF (*n* = 52)	*P*-value
GLSA4C	−14.45 (−19.95 ∼ −8.8)	−11.4 (−16.6 ∼ −5.9)	−18.4 (−24.57 ∼ −11. 1)	<0.001
GLSA2C	−14.30 (−20.82 ∼ −8.82)	−9.75 (−15.92 ∼ −6.55)	−16.55 (−26.05 ∼ −12.52)	<0.001
GLSA3C	−13.75 (−20.97 ∼ −8.5)	−9.65 (−14.37 ∼ −5.05)	−17.6 (−25.25 ∼ −12.87)	<0.001
LV-GLS	−13.8 (−20.32 ∼ −9.37)	−10.4 (−14.4 ∼ −6.2)	−18.15 (−24.47 ∼ −12.45)	<0.001
LASr-ED	18.3 (9.9 ∼ 30. 17)	11.65 (6 ∼ 21.85)	24.2 (13.62 ∼ 35.65)	<0.001
LASrcd-ED	−8.6 (−12.95 ∼ −4.9)	−6.3 (−11.2 ∼ −3.75)	−11.4 (−18.6 ∼ −7. 17)	<0.001
LASrct-ED	−9.5 (−16.07 ∼ −3.3)	−5.85 (−12.5 ∼ −2.07)	−13.35 (−19.42 ∼ −6.7)	0.002
LAFI	3.43 (2.22 ∼ 8.48)	5.4 (2.92 ∼ 12.51)	2.71 (1.82 ∼ 5.47)	0.005
LASI	0.78 (0.30 ∼ 1.59)	1.08 (0.59 ∼ 2.45)	0.42 (0.23 ∼ 1.06)	<0.01
RVFWSL	−17.75 (−23.22 ∼ −13.57)	−17.39 (−20.27 ∼ −11.3)	−20.2 (−26.02 ∼ −14.8)	0.025
RV4CSL	−15.12 (−18.75 ∼ −11)	−12.5 (−16.35 ∼ −7.62)	−17 (−20.42 ∼ −12.05)	<0.001

### 4.4 Correlation between LVEF and 2D speckle tracking parameters in different heart failure phenotypes

**Figure 1:**
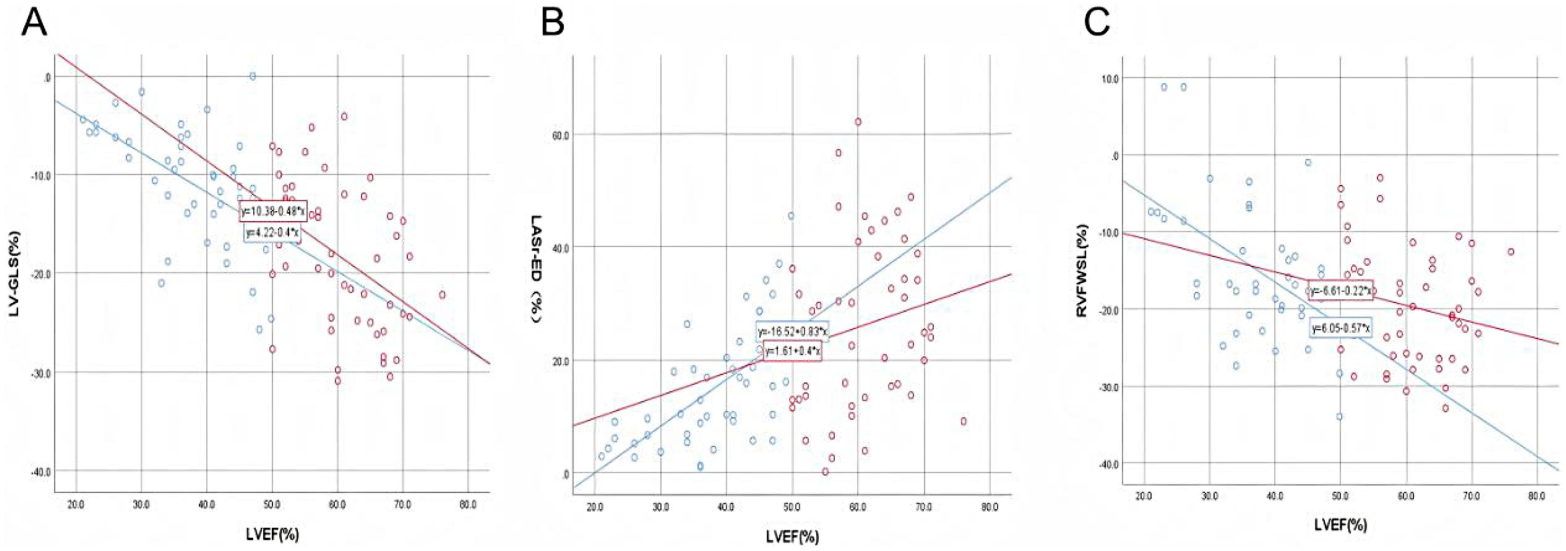
Correlations between LVEF and myocardial strain parameters in patients with different heart failure phenotypes. Scatter plots showing the relationships between LVEF and LV-GLS **(A)**, LASr **(B)**, and RVFWLS **(C)** in patients with HFrEF and HFpEF. Blue dots and regression lines represent the HFrEF group, while red dots and regression lines represent the HFpEF group. Linear regression lines are shown for each group. LV-GLS, left ventricular global longitudinal strain; LASr, left atrial reservoir strain; RVFWLS, right ventricular free wall longitudinal strain; LVEF, left ventricular ejection fraction; HFrEF, heart failure with reduced ejection fraction; HFpEF, heart failure with preserved ejection fraction.

In the correlation analysis of 2D speckle tracking parameters and LVEF in different heart failure phenotypes, the results showed that in the HFrEF subgroup, LVEF was negatively correlated with GLSA4C, GLSA2C, GLSA3C, LV-GLS, RVFWSL, and RV4CSL (*P* < 0.05) (Figure 1). Additionally, LVEF was negatively correlated with LASr-ED, LASrct-ED, LAFI, and LASI (*P* < 0.05), but positively correlated with LASrcd-ED. In the HFpEF subgroup, LVEF was only negatively correlated with GLSA4C, GLSA2C, GLSA3C, and LV-GLS (*P* < 0.05).

### 4.5 Multivariable linear regression analyses for LASr-ED

As shown in Table 5, multivariable linear regression analysis was performed to evaluate the independent association between LV-GLS and LASr-ED in different HF phenotypes. After adjustment for LVEF, SBP, and NT-proBNP, LV-GLS remained independently associated with LASr-ED in both the HFrEF and HFpEF subgroups. In the HFrEF subgroup, LV-GLS was significantly associated with LASr-ED (B =  −0.889, 95% CI −1.367 to −0.411). A similar association was observed in the HFpEF subgroup (B =  −0.952, 95% CI −1.536 to −0.368).

### 4.6 Correlation between right and left ventricular strain and left atrial strain parameters in different heart failure phenotypes

**Figure 2:**
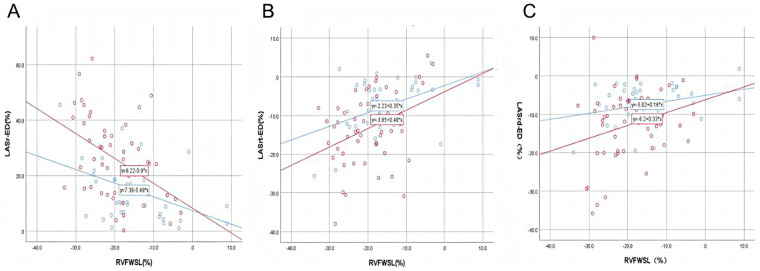
Correlations between RVFWLS and left atrial strain parameters in patients with HFrEF and HFpEF. Scatter plots with fitted linear regression lines illustrate the associations between RVFWLS and left atrial strain parameters in the HFrEF and HFpEF groups. **(A)** Association between RVFWLS and LASr-ED. **(B)** Association between RVFWLS and LASct-ED. **(C)** Association between RVFWLS and LAScd-ED. Blue dots and regression lines represent the HFrEF group, while red dots and regression lines represent the HFpEF group. HFrEF, heart failure with reduced ejection fraction; HFpEF, heart failure with preserved ejection fraction; RVFWLS, right ventricular free wall longitudinal strain; LASr-ED, left atrial reservoir strain at end-diastole; LASct-ED, left atrial contraction strain at end-diastole; LAScd-ED, left atrial conduit strain at end-diastole.

From the figures, we observe the following correlations:In both groups of heart failure patients, RVFWSL is negatively correlated with LASr-ED (*P* < 0.05) and positively correlated with LASrct-ED (*P* < 0.05), but its correlation with LASrcd-ED disappears (*P* > 0.05); LV-GLS is negatively correlated with LASr-ED (*P* < 0.05) and positively correlated with LASrcd-ED and LASrct-ED (*P* < 0.05) (Figure 2). From the results in Table 4, we find that after controlling for the related variable (LVEF). In both groups of heart failure patients, the correlations between LV-GLS and LASr-ED, LASrcd-ED, and LASrct-ED remain significant (*P* < 0.05); In the HFrEF group, the correlations between RVFWSL and LASr-ED, LASrcd-ED, and LASrct-ED disappear (*P* > 0.05). In the HFpEF group, RVFWSL remains negatively correlated with LASr-ED (*P* < 0.05) and positively correlated with LASrct-ED (*P* < 0.05), but its correlation with LASrcd-ED disappears (*P* > 0.05). Details are presented in Table 6.

**Table 4:** Correlation between LVEF and 2D-STE parameters in different heart failure phenotypes.

Variables	HFrEF		HFpEF	
	*r*	*P*	*r*	*P*
GLSA4C	−0.496	<0.001	−0.437	0.001
GLSA2C	−0.592	<0.001	−0.410	0.003
GLSA3C	−0.458	<0.001	−0.464	<0.001
LV-GLS	−0.580	<0.001	−0.471	<0.001
LASr-ED	0.648	<0.001	0.233	0.097
LASrcd-ED	−0.392	0.007	−0.246	0.079
LASrct-ED	−0.650	<0.001	−0.181	0.2
LAFI	−0.49	<0.001	−0.182	0.197
LASI	−0.394	0.007	−0.207	0.142
RVFWSL	−0.409	0.005	−0.194	0.167
RV4CSL	−0.449	0.002	−0.233	0.096

**Table 5:** Multivariable linear regression analyses for LASr-ED.

Dependent variable	Predictor	HFrEF		HFpEF	
		B (95% CI)	*P*	B (95% CI)	*P*
LASr-ED	LVEF	0.480 (0.095, 0.865)	0.016	−0.146 (−0.729, 0.437)	0.616
	SBP	−0.046 (−0.189, 0.097)	0.519	−0.019 (−0.201, 0.163)	0.832
	NT-proBNP	0.000 (−0.001, 0.000)	0.030	−0.001 (−0.002, 0.000)	0.054
	LV-GLS	−0.889 (−1.367, −0.411)	0.001	−0.952 (−1.536, −0.368)	0.002

**Table 6:** Correlation between right and left ventricular strain and left atrial strain parameters in different heart failure phenotypes.

Predictor	Left atrial strain parameter	HFrEF r	HFrEF P	HFpEF r	HFpEF P
RVFWSL	LASr-ED	−0.339	0.021	−0.455	<0.001
	LASrcd-ED	0.127	0.4	0.271	0.052
	LASrct-ED	0.362	0.013	0.371	0.007
LV-GLS	LASr-ED	−0.638	<0.001	−0.528	<0.001
	LASrcd-ED	0.497	<0.001	0.449	<0.001
	LASrct-ED	0.578	<0.001	0.399	0.003
Controlling for LVEF					
RVFWSL	LASr-ED	−0.102	0.503	−0.431	0.002
	LASrcd-ED	0.021	0.891	0.237	0.094
	LASrct-ED	0.162	0.289	0.347	0.013
LV-GLS	LASr-ED	−0.499	<0.001	−0.502	<0.001
	LASrcd-ED	0.431	0.003	0.405	0.003
	LASrct-ED	0.359	0.015	0.365	0.009

## 5 Discussion

ADHF is a highly heterogeneous clinical syndrome driven by complex pathophysiological mechanisms. Its pathogenesis involves a dynamic interplay of biventricular systolic and diastolic dysfunction, altered arterial and venous tone, neurohormonal and inflammatory activation, and the cumulative burden of comorbidities (2).

In this study, 98 patients with ADHF were divided into two groups based on theirEF: HFrEF and HFpEF. Through statistical analysis of 2D speckle-tracking echocardiographic parameters, we found significant differences in the speckle-tracking indices between the HFrEF and HFpEF groups (*P* < 0.05), and EF showed a correlation with these speckle-tracking parameters (*P* < 0.05). After adjusting for EF, LV-GLS remained correlated with various indices in the different heart failure groups. Our findings suggest a stronger interdependence between right ventricular and left atrial mechanics in HFpEF patients compared to HFrEF patients, evidenced by the after adjusting for LVEF.

### 5.1 Left ventricular function evaluation

Global longitudinal strain (GLS) is an emerging cardiac biomarker, with an increasing body of research indicating that GLS can serve as a prognostic marker and risk stratification tool for heart failure patients (20–24). Sven-Oliver et al. (9) found that GLS is associated with cardiac mortality and all-cause mortality in heart failure patients, independent of clinical condition and cardiac structure or function.

Importantly, GLS remains associated with cardiac mortality even after additional adjustments for standard biomarkers like NT-proBNP, suggesting that it may improve risk stratification and management of heart failure patients. In this study, we observed that LV-GLS was reduced in HFrEF patients, significantly lower than in HFpEF patients. In the HFpEF group, GLS values less than |−16| were considered abnormal, and we found that 30 patients (57.7%) had GLS <|−16|. This result aligns with studies by Jurica et al. and Bshiebish et al., who also found reduced LV-GLS in both HFrEF and HFpEF patients, with a more pronounced reduction in HFrEF. This may be explained by the positioning of longitudinal fibers in the subendocardial layer in HFpEF, making it more susceptible to ischemia, ventricular hypertrophy, and systolic-diastolic abnormalities. LAFI can detect left ventricular systolic dysfunction (the reduction in longitudinal contractile force), LV-GLS is a better indicator of subtle differences in left ventricular performance than LVEF. The decrease in GLS indicates impaired systolic function ，in our study, the GLS of the HFpEF group was less than |−16|. These patients were characterized by the following: Lower LVEF [63.5 (59–68) vs. 55.5 (51.7–61), *P* < 0.005], Higher SUA [361. 1 ± 125.412 vs. 454.64 ± 144.655, *P* < 0.005], Lower eGFR [80.55 (61.35–101.03) vs. 70.81 (49.19–84.26), *P* < 0.005], Larger left ventricular end-systolic diameter [32.23 ± 4.897 vs36.77 ± 5.814, *P* < 0.005], Higher BMI [23.564 (21.18–25.90) vs. 26.190 (22.61–28.35), *P* < 0.005], Lower LASr_ED: [31.967 ± 13.499 vs. 17.391 ± 11.157, *P* < 0.001], Lower LASrd_ED [−16.06 ± 8.943 vs. −8.191 ± 6.989, *P* < 0.05], Lower LASrt_ED [−16.233 ± 7.964 vs.−8.941 ± 9.623, *P* < 0.05], Lower RVFWSL1 [−21.817 ± 6.180 vs. −16.526 ± 7.736, *P* < 0.05], Larger right atrial diameter [34 (29–37) vs. 37 (31.5–40), *P* < 0.05].

Accurate evaluation of left ventricular systolic function is crucial for diagnosing and treating cardiovascular diseases. LVEF and GLS are the most important indicators of cardiac systolic function. LVEF is a primary indicator of systolic function but is less sensitive to early ventricular systolic dysfunction. The ESC Heart Failure Association's guidelines suggest that GLS is superior to LVEF in evaluating subclinical systolic dysfunction, given its stability and reproducibility. In our study, we found a negative correlation between LVEF and GLS in both groups of heart failure patients (*P* < 0.005), further indicating that GLS reflects overall left ventricular myocardial contractile changes. This analysis was performed not merely to confirm the known LVEF-GLS relationship, but to explore how the coupling between LV function (as measured by LVEF) and other cardiac chambers (LA, RV) differs between HFrEF and HFpEF. The finding that LVEF correlates with RV and LA strain only in HFrEF, but not in HFpEF, highlights the potential for RV and LA strain to provide unique insights into the pathophysiology of HFpEF that are independent of conventional LVEF. The study by Pibarot et al. suggests that in patients with severe aortic stenosis (AS) and normal LVEF, the thicker the ventricular wall, the more severe the impairment of longitudinal systolic function. This may be related to the anatomical characteristics of myocardial fibers, as longitudinal fibers dominate the myocardium and are more sensitive to endocardial ischemia. Moreover, compensatory myocardial remodeling becomes increasingly pronounced. In a study of acute decompensated myocardial infarction patients, GLS demonstrated superior prognostic value compared to LVEF, early diastolic flow velocity peak (E), and the E/E′ ratio. In a small study involving 54 hospitalized patients with heart failure and an LVEF greater than 45%, GLS indicated an increased risk of all-cause mortality when adjusted with a cutoff level of −15.8% (25). Additionally, GLS was found to independently predict poor long-term outcomes in both HFrEF and HFpEF patients.

### 5.2 Left atrial function evaluation

As the research further develops, more and more evidence suggests that the left atrium plays a critical role in maintaining heart function and during the entire cardiac cycle. Physiologically, the left atrium serves as a reservoir, conduit, and pump. The reservoir function refers to the collection of pulmonary venous return during left ventricular systole (measured by LASr); the conduit function refers to the passage of blood from the pulmonary veins to the left ventricle during early diastole (measured by LAScd); and the pump function refers to the active contraction of the left atrium during late diastole (measured by LASct). The close relationship between left atrial function and overall heart function makes the study of left atrial changes significant for diagnosis and treatment. In this study, ADHF patients were divided into two groups based on ejection fraction. Significant differences in LASr, LAScd, and LASct values were observed between the groups. The absolute values of LASr, LAScd, and LASct in the HFrEF group were lower than those in the HFpEF group, indicating that left atrial function was more severely impaired in HFrEF patients compared to those with HFpEF. Our results showed that the median LASr in the HFpEF group was 24.2% (13.625%–35.650%), the median LAScd was −11.4% (−18.6%–7. 175%), and the median LASct was −13.350% (−19.425%–−6.7%). A meta-analysis involving 39,982 patients suggested that the normal values for LASr, LAScd, and LASct are 39.4%, −23%, and −17.4% (26). This indicates that while left ventricular systolic function in the HFpEF group remains within the normal range, the reservoir, conduit, and pump functions of the left atrium (LA) are significantly impaired. LA strain parameters are superior to conventional echocardiographic LVEF in detecting early left heart dysfunction (27). LA reservoir and pump functions have been shown to be impaired in both HFpEF and HFrEF (28). Kurt et al. (29) found that LA strain during LV systole, measured using TDE-based strain imaging, was significantly lower in HFpEF patients compared to LV diastolic dysfunction (DD) patients without HF. The LA reservoir and booster pump functions in HFrEF patients were significantly worse than those in HFpEF patients, consistent with our study findings. Additionally, in our analysis, LASr, LAScd, and LASct were correlated with LVEF only in HFrEF patients, but no such correlation was found in HFpEF patients. This suggests that minor pathological changes in LA function in HFpEF patients may have less impact on LVEF, whereas in HFrEF, LA dysfunction may exacerbate heart failure, resulting in a lower LVEF. Population-based studies have demonstrated that LA reservoir dysfunction exists in HF patients with reduced ejection fraction (30, 31) and in patients with stable coronary artery disease, serving as a predictor of cardiovascular outcomes (32, 33). A systematic review and meta-analysis of 61 studies compared LA structural and functional changes in 8,806 HF-rEF and 9,928 HF-pEF patients. The pooled data showed that both in the acute and chronic phases, HF-rEF patients had worse LAS-r compared to HF-pEF patients (acute: 9.0% vs. 18.9%; chronic: 12.8% vs. 23.4%) (34). Reduced LAS-r is associated with adverse events and can serve as an important long-term prognostic predictor independent of other clinical and echocardiographic parameters, such as LA volume, LV longitudinal strain (LVLS), age, LV-EF, and E/E′ ratio (35, 36). In acute heart failure, a large study of 4,312 patients demonstrated that LAS-r ≤ 18% is an independent predictor of new-onset atrial fibrillation over a 5-year follow-up, regardless of whether patients had HF-rEF or HF-mrEF (37). In acute heart failure patients with sinus rhythm, LAS-r < 14.5% was an independent predictor of stroke, associated with a 2.38% annual increase in stroke incidence (38).

In both groups of heart failure patients, RVFWSL was negatively correlated with LASr-ED (*P* < 0.05) and positively correlated with LASrct-ED. However, after controlling for ejection fraction, we found that RVFWSL was correlated with LASr-ED and LASrct-ED only in HFpEF patients, but not in HFrEF patients. A possible explanation is that in the early stages, LA reservoir and pump functions increase as compensatory mechanisms, while LA conduit function (peak early diastolic forward flow velocity) decreases. As heart failure progresses, LA conduit function increases, LA reservoir function decreases, and pump function significantly increases. With further deterioration of atrial contractile function, LA conduit function becomes dominant, while LA reservoir and pump functions are severely impaired, corresponding to marked LV dysfunction. LAS can detect subtle changes in LA reservoir, conduit, and pump functions at various stages of heart failure development, even before LA volume changes (39, 40).

Our study also observed a correlation between LA function and GLS. After controlling for LVEF as a covariate, the LA-LV coupling relationship was still evident in both heart failure groups. This suggests that GLS is an important influencing factor for LA reservoir and systolic strain, consistent with previous research. Sun et al. (41) demonstrated a significant correlation between LA strain and GLS. Additionally, other studies have shown that LASr and LAScd reduce,and LA-LV coupling relationship is abnormal in diabetic patients (42). This indicates a close relationship between reduced LA function and LV dysfunction. Changes in LA function may partially result from LV dysfunction, as increased LA afterload is likely caused by LV dysfunction and abnormalities in the LA-LV coupling relationship.

In HFpEF patients, the left atrial function index (LAFI) demonstrates good diagnostic performance for identifying elevated LV filling pressure (cutoff value: 2.7; sensitivity: 74.2%; specificity: 94.4%). Furthermore, it is highly effective in classifying diastolic dysfunction and accurately diagnosing HFpEF, especially in patients with an “indeterminate” status (43). In our study, we found significant differences in LAFI between HFrEF and HFpEF patients. However, correlation analysis revealed that LAFI was negatively correlated with LVEF only in HFrEF patients, with no significant correlation observed in HFpEF patients. This finding is consistent with Anthony's study (42), which also reported a correlation between LAFI and ejection fraction (*r* = 0.616, *P* = 0.001). Similarly, another study (44) noted that as LAFI increased, ejection fraction significantly improved. The relationship between LA function and ejection fraction further highlights that as the ejection fraction decreases in heart failure patients, LA function is also affected, and vice versa. In other words, these parameters influence each other, suggesting that the left atrial function index can serve as a biomarker for left ventricular ejection fraction and vice versa.

Kurt et al. (29) attempted to use the ratio of E/e′ to LASr as a substitute for the ratio of left atrial pressure (LAP) to peak atrial longitudinal strain (PALS) to describe the left atrial stiffness index (LASI). They found that, compared with the control group, HFpEF patients exhibited reduced LASr and increased LASI using both measurement methods. Porpácz et al. (45) reported that LASI predicts elevated left ventricular filling pressure (defined as NT-proBNP >220 pg/mL) more effectively than the left atrial volume index (LAVI) and LASr, emphasizing the critical role of atrial mechanics in the accurate risk stratification of HFpEF patients. This concept was further supported by Obokata et al. (46) who demonstrated that LA strain is a better predictor than conventional echocardiographic parameters. Kurt et al. (29) also showed that LASI, calculated as the ratio of LV filling pressure estimated during LV systole to LV strain, is the most accurate indicator for differentiating HFpEF patients from those with diastolic dysfunction (DD). These findings are consistent with our study results. However, during subgroup analysis, we found that LASI correlated with LVEF only in HFrEF patients, with a negative correlation. We hypothesize that changes in LASI have a greater impact on LVEF in HFrEF patients.

In fact, the LA and LV are closely related anatomically (via the basal ring) and functionally (“in tandem”). Consequently, LA function is inherently influenced by LV systolic performance, creating a tethering effect that enhances or facilitates LV passive filling (reservoir phase) and diastole, which ultimately contributes to mitral valve filling during the passive “conduit” phase of the cardiac cycle. In recent years, multiple studies have shown that LA strain is impaired in patients with LV diastolic dysfunction and correlates well with LV filling pressure (LVFP) or pulmonary capillary wedge pressure (PCWP) (29, 43, 47, 48). Therefore, the LA filling index, defined as the ratio of mitral E velocity to LASr, reflects changes in LA function (LASr) and the current LV filling status (mitral E velocity). The left atrial stiffness index, which incorporates optimal LV strain parameters and superior LVFP indicators (E/e′), can capture the influence of LV diastolic function on LA compliance and the impact of LA function on LV diastolic performance. Thus, the LA filling index, LASI, and LAVI/LASr may serve as potential indicators for detecting elevated LVFP (46).

### 5.3 Right ventricular function evaluation

Common right ventricular (RV) strain parameters, such as RV global longitudinal strain (RVGLS) and RV free wall strain (RVFWS), are useful for assessing right ventricular function. RVGLS represents the average strain from the interventricular septum and right ventricular free wall, while RVFWS represents the strain of the free wall alone. However, RVGLS is influenced by left ventricular motion, which can reduce its accuracy. Therefore, RVFWS is recommended for evaluating right ventricular longitudinal strain. RV strain analysis is technically challenging, but 2D-STE provides a useful tool to overcome limitations in conventional assessment.

According to Iacoviello et al., RV strain parameters, including RVFWSL and RV4CSL, are significantly correlated with LVEF, the E/e′ ratio, and NYHA classification. Additionally, both are significantly correlated with other indices of RV systolic function (49). These findings are similar to our own. In our study, RVFWSL and RV4CSL were correlated with LVEF. However, upon performing correlation analysis, we found that only in HFrEF patients did RVFWSL and RV4CSL correlate negatively with LVEF. In contrast, in HFpEF patients, neither RVFWSL (*P* = 0. 167) nor RV4CSL (*P* = 0.096) showed significant correlation with LVEF. We speculate that this may be due to HFpEF being in the early stages of heart failure, where pulmonary congestion is less severe, pulmonary artery pressure is not significantly elevated, and the impact on right heart function is less pronounced. On the other hand, in HFrEF, heart failure is at a later stage, with significantly elevated pulmonary artery pressure, which in turn affects right ventricular function. This suggests that RV strain may be particularly useful in our clinical practice, providing additional clinical significance. In current research, RVFWSL is correlated with both systolic and diastolic pressures of the left ventricle, as well as with conventional left ventricular function parameters. The relationship between RV and LV systolic and diastolic function is well-established and supports the concept of ventricular interdependence. RV strain measurements may be more sensitive than traditional echocardiographic parameters in detecting subtle myocardial dysfunction, which is consistent with scientific reports (50–53). Although cardiac magnetic resonance imaging (MRI) is the gold standard for non-invasive assessment of RV size and function, it is time-consuming, expensive, and often impractical. Therefore, RV strain, as a highly sensitive 2D echocardiographic marker of RV contractility, is relatively easy to obtain and less demanding. In routine clinical practice, it may play a crucial role in the comprehensive evaluation of RV function.

## 6 Innovation and limitations

There are several limitations in this study. First, the sample size of this study is relatively small, partly because 2D strain requires precise measurements and appropriate images from A4C, A3C, and A2C projections. Therefore, our results may only be applicable toADHF patients with good echocardiographic image quality for 2D strain analysis and may not be generalizable to all patients. Second, the absence of a separate HFmrEF analysis is a significant limitation of our study. The small number of patients in this category (*n* = 8) prevented a meaningful three-group comparison. Future studies with larger, more diverse cohorts are warranted to investigate the specific strain characteristics of the HFmrEF phenotype. Third, the timing of echocardiography within the first 72 h of admission, without strict standardization relative to clinical decongestion or specific therapeutic interventions, represents a significant potential confound. Changes in preload and afterload during this period could influence strain values. Information on the patients' exact location (e.g., emergency department vs. ward) and concurrent medical therapies at the time of the echo was not available for analysis. Future studies should ideally acquire strain data at a pre-specified time point, such as at the time of clinical near-euvolemia, to improve the reliability of the findings. Fourth, heart rate at the time of the echocardiogram was not included in our analysis. As strain rates are intrinsically linked to the cardiac cycle duration, the inability to adjust for this variable is a weakness. This should be considered in the design of future prospective studies. Fifth, the retrospective nature of this study, which, due to the lack of long-term follow-up, prevents us from establishing a causal relationship between speckle tracking and short-term or long-term prognosis in ADHF. Sixth, not all measurements were made by a single cardiologist (but by two individuals), which introduces unavoidable inter-observer variability in the recording and interpretation of echocardiographic data. However, the 2D strain analysis was conducted by one person to minimize the risk of inter-observer variability in this analysis. Seventh, the presence of various subtypes of HFpEF may have contributed to negative results. Last, HFrEF often involves multiple comorbidities such as dilated cardiomyopathy, diabetes, and renal insufficiency, which may also lead to negative results. Despite these limitations, our study contributes to the growing body of evidence that 2D_ STE parameters, particularly LV_GLS, may offer additional insights beyond LVEF in characterizing ADHF phenotypes. Whether these parameters can guide clinical management in ADHF remains to be determined in future prospective studies.

## 7 Conclusion

Our study shows that compared to the HFrEF group, the HFpEF group exhibits significant differences in speckle tracking parameters. Ejection fraction is correlated with speckle tracking indices, and after adjusting for ejection fraction, LV-GLS remains correlated with other indices in both heart failure groups. Our findings suggest a stronger interdependence between right ventricular and left atrial mechanics in HFpEF patients compared to HFrEF patients, evidenced by the persistence of correlations between RV free wall strain and LA reservoir/pump strain after adjusting for LVEF.

## Data Availability

The raw data supporting the conclusions of this article will be made available by the authors, without undue reservation.

## References

[1] Yancy CW Jessup M Bozkurt B Butler J Casey DE Colvin MM. 2017 ACC/AHA/HFSA focused update of the 2013 ACCF/AHA guideline for the management of heart failure: a report of the American college of cardiology/American heart association task force on clinical practice guidelines and the heart failure society of America. J Am Coll Cardiol. (2017) 70(6):776–803. 10.1016/j.jacc.2017.04.025 28461007

[2] Njoroge JN Teerlink JR Teerlink JR. Pathophysiology and therapeutic approaches to acute decompensated heart failure. Circ Res. (2021) 128(10):1468–86. 10.1161/CIRCRESAHA.121.318186 33983837 PMC8126502

[3] Brann A Miller J Eshraghian E Park JJ Park JJ. Global longitudinal strain predicts clinical outcomes in patients with heart failure with preserved ejection fraction. Eur J Heart Fail. (2023) 25(10):1755–65. 10.1002/ejhf.2947 37369633

[4] Potter E Marwick TH. Assessment of left ventricular function by echocardiography: the case for routinely adding global longitudinal strain to ejection fraction. JACC Cardiovasc Imaging. (2018) 11(2 Pt 1):260–74. 10.1016/j.jcmg.2017.11.017 29413646

[5] Hashemi D Motzkus L Blum M Kraft R Tanacli R Tahirovic E. Myocardial deformation assessed among heart failure entities by cardiovascular magnetic resonance imaging. ESC Heart Fail. (2021) 8(2):890–7. 10.1002/ehf2.13193 33539681 PMC8006725

[6] Voigt J-U Cvijic M. 2- and 3-dimensional myocardial strain in cardiac health and disease. JACC Cardiovasc Imaging. (2019) 12(9):1849–63. 10.1016/j.jcmg.2019.01.044 31488253

[7] Marwick TH Shah SJ Shah SJ Thomas JD. Myocardial strain in the assessment of patients with heart failure: a review. JAMA Cardiol. (2019) 4(3):287–94. 10.1001/jamacardio.2019.0052 30810702

[8] Donal E Neveu A Fontes-Carvalho R Fontes-Carvalho R. Global longitudinal strain: ready for ‘prime time’ in heart failure characterization. Eur J Heart Fail. (2023) 25(10):1766–7. 10.1002/ejhf.3012 37634948

[9] Tröbs S-O Prochaska JH Schwuchow-Thonke S Schulz A Müller F Heidorn MW. Association of global longitudinal strain with clinical Status and mortality in patients with chronic heart failure. JAMA Cardiol. (2021) 6(4):448–56. 10.1001/jamacardio.2020.7184 33533883 PMC7859875

[10] Mignot A Donal E Zaroui A Reant P Salem A Hamon C. Global longitudinal strain as a major predictor of cardiac events in patients with depressed left ventricular function: a multicenter study. J Am Soc Echocardiogr. (2010) 23(10):1019–24. 10.1016/j.echo.2010.07.019 20810243

[11] Stanton T Leano R Marwick TH. Prediction of all-cause mortality from global longitudinal speckle strain: comparison with ejection fraction and wall motion scoring. Circ Cardiovasc Imaging. (2009) 2(5):356–64. 10.1161/CIRCIMAGING.109.862334 19808623

[12] Witkowski TG Thomas JD Debonnaire PJMR Delgado V Hoke U Ewe SH. Global longitudinal strain predicts left ventricular dysfunction after mitral valve repair. Eur Heart J Cardiovasc Imaging. (2013) 14(1):69–76. 10.1093/ehjci/jes155 22848021

[13] Holland DJ Marwick TH Haluska BA Leano R Hordern MD Hare JL. Subclinical LV dysfunction and 10-year outcomes in type 2 diabetes mellitus. Heart. (2015) 101(13):1061–6. 10.1136/heartjnl-2014-307391 25935767

[14] Nogi S Ito T Kizawa S Shimamoto S Sohmiya K Hoshiga M. Association between left ventricular postsystolic shortening and diastolic relaxation in asymptomatic patients with systemic hypertension. Echocardiography (Mount Kisco, N.Y.). (2016) 33(2):216–22. 10.1111/echo.13022 26234318

[15] Halabi A Yang H Wright L Potter E Huynh Q Negishi K. Evolution of myocardial dysfunction in asymptomatic patients at risk of heart failure. JACC Cardiovasc Imaging. (2021) 14(2):350–61. 10.1016/j.jcmg.2020.09.032 33221236

[16] Paldino A De Angelis G Dal Ferro M Faganello G Porcari A Barbati G. High prevalence of subtle systolic and diastolic dysfunction in genotype-positive phenotype-negative relatives of dilated cardiomyopathy patients. Int J Cardiol. (2021) 324:108–14. 10.1016/j.ijcard.2020.09.036 32949639

[17] Cirino RHD Scola RH Ducci RD-P Wermelinger ACC Kay CSK Lorenzoni PJ. Predictors of early left ventricular systolic dysfunction in duchenne muscular dystrophy patients. Muscle Nerve. (2018) 58(1):84–9. 10.1002/mus.26102 29443387

[18] Yang H Negishi K Wang Y Nolan M Saito M Marwick TH. Echocardiographic screening for non-ischaemic stage B heart failure in the community. Eur J Heart Fail. (2016) 18(11):1331–9. 10.1002/ejhf.643 27813300

[19] McDonagh TA Metra M Adamo M Gardner RS Baumbach A Böhm M. 2021 ESC guidelines for the diagnosis and treatment of acute and chronic heart failure. Eur Heart J. (2021) 42(36):3599–726. 10.1093/eurheartj/ehab368 34447992

[20] Jurica J Péč MJ Cingel M Bolek T Barbierik Vachalcová M Horná S. Left ventricular and atrial deformation in patients with acute decompensated heart failure: a pilot study. Diagnostics (Basel, Switzerland). (2024) 14(13):1368. 10.3390/diagnostics14131368 39001258 PMC11240885

[21] Bshiebish HAH Al-Musawi AH Khudeir SA. Role of global longitudinal strain in assessment of left ventricular systolic function in patients with heart failure with preserved ejection fraction. J Saudi Heart Assoc. (2019) 31(2):100–5. 10.1016/j.jsha.2018.12.002 30766004 PMC6360320

[22] Slivnick JA Singulane C Sun D Eshun D Narang A Mazzone S. Preservation of circumferential and radial left ventricular function as a mitigating mechanism for impaired longitudinal strain in early cardiac amyloidosis. J Am Soc Echocardiogr. (2023) 36(12):1290–301. 10.1016/j.echo.2023.08.005 37574149

[23] Marwick TH Leano RL Brown J Sun J-P Hoffmann R Lysyansky P. Myocardial strain measurement with 2-dimensional speckle-tracking echocardiography: definition of normal range. JACC Cardiovasc Imaging. (2009) 2(1):80–4. 10.1016/j.jcmg.2007.12.007 19356538

[24] Huang W Chai SC Lee SGS MacDonald MR Leong KTG. Prognostic factors after index hospitalization for heart failure with preserved ejection fraction. Am J Cardiol. (2017) 119(12):2017–20. 10.1016/j.amjcard.2017.03.032 28477861

[25] Sengeløv M Jørgensen PG Jensen JS Bruun NE Olsen FJ Fritz-Hansen T. Global longitudinal strain is a superior predictor of all-cause mortality in heart failure with reduced ejection fraction. JACC Cardiovasc Imaging. (2015) 8(12):1351–9. 10.1016/j.jcmg.2015.07.013 26577264

[26] Shah KS Xu H Matsouaka RA Bhatt DL Heidenreich PA Hernandez AF. Heart failure with preserved, borderline, and reduced ejection fraction: 5-year outcomes. J Am Coll Cardiol. (2017) 70(20):2476–86. 10.1016/j.jacc.2017.08.074 29141781

[27] Park J-H Hwang I-C Park JJ Park J-B Cho G-Y. Prognostic power of left atrial strain in patients with acute heart failure. Eur Heart J Cardiovasc Imaging. (2021) 22(2):210–9. 10.1093/ehjci/jeaa013 32031588

[28] Vieira MJ Teixeira R Gonçalves L Gersh BJ. Left atrial mechanics: echocardiographic assessment and clinical implications. J Am Soc Echocardiogr. (2014) 27(5):463–78. 10.1016/j.echo.2014.01.021 24656882

[29] Kurt M Wang J Torre-Amione G Nagueh SF. Left atrial function in diastolic heart failure. Circ Cardiovasc Imaging. (2009) 2(1):10–5. 10.1161/CIRCIMAGING.108.813071 19808559

[30] Morris DA Belyavskiy E Aravind-Kumar R Kropf M Frydas A Braunauer K. Potential usefulness and clinical relevance of adding left atrial strain to left atrial volume index in the detection of left ventricular diastolic dysfunction. JACC Cardiovasc Imaging. (2018) 11:1405–15. 10.1016/j.jcmg.2017.07.029 29153567

[31] Potter EL Ramkumar S Kawakami H Yang H Wright L Negishi T. Association of asymptomatic diastolic dysfunction assessed by left atrial strain with incident heart failure. JACC Cardiovasc Imaging. (2020) 13:2316–26. 10.1016/j.jcmg.2020.04.028 32771583

[32] Santos ABS Kraigher-Krainer E Gupta DK Claggett B Zile MR Pieske B. Impaired left atrial function in heart failure with preserved ejection fraction. Eur J Heart Fail. (2014) 16:1096–103. 10.1002/ejhf.147 25138249 PMC5535768

[33] Sanchis L Gabrielli L Andrea R Falces C Duchateau N Perez-Villa F. Left atrial dysfunction relates to symptom onset in patients with heart failure and preserved left ventricular ejection fraction. Eur Heart J Cardiovasc Imaging. (2015) 16:62–7. 10.1093/ehjci/jeu165 25187609

[34] Jin X Nauta JF Hung C-L Ouwerkerk W Teng T-HK Voors AA. Left atrial structure and function in heart failure with reduced (HFrEF) versus preserved ejection fraction (HFpEF): systematic review and meta-analysis. Heart Fail Rev. (2022) 27:1933–55. 10.1007/s10741-021-10204-8 35079942 PMC9388424

[35] Carluccio E Biagioli P Mengoni A Francesca Cerasa M Lauciello R Zuchi C. Left atrial reservoir function and outcome in heart failure with reduced ejection fraction. Circ Cardiovasc Imaging. (2018) 11:e007696. 10.1161/CIRCIMAGING.118.007696 30571318

[36] Chimed S Stassen J Galloo X Meucci MC Van Der Bijl P Marsan NA. Left atrial reservoir strain and long-term prognosis in patients with heart failure and reduced ejection fraction. Eur Heart J. (2022) 43(Suppl 2):ehac544.926. 10.1093/eurheartj/ehac544.926

[37] Park JJ Park JH Hwang IC Park JB Cho GY Marwick TH. Left atrial strain as a predictor of new-onset atrial fibrillation in patients with heart failure. JACC Cardiovasc Imaging. (2020) 13:2071–81. 10.1016/j.jcmg.2020.04.031 32682715

[38] Park JH Hwang IC Park JJ Park JB Cho GY. Left atrial strain to predict stroke in patients with acute heart failure and sinus rhythm. J Am Heart Assoc. (2021) 10:e020414. 10.1161/JAHA.120.020414 34187174 PMC8403314

[39] Al Saikhan L Hughes AD Chung WS Alsharqi M Nihoyannopoulos P. Left atrial function in heart failure with mid-range ejection fraction differs from that of heart failure with preserved ejection fraction: a 2D speckle-tracking echocardiographic study. Eur Heart J Cardiovasc Imaging. (2019) 20:279–90. 10.1093/ehjci/jey171 30517648 PMC6383056

[40] Frydas A Morris DA Belyavskiy E Radhakrishnan A-K Kropf M Tadic M. Left atrial strain as sensitive marker of left ventricular diastolic dysfunction in heart failure. ESC Heart Fail. (2020) 7:1956–65. 10.1002/ehf2.12820 32613770 PMC7373910

[41] Sun BJ Park J-H Lee M Choi J-O Lee J-H Shin M-S. Normal reference values for left atrial strain and its determinants from a large Korean multicenter registry. J Cardiovasc Imaging. (2020) 28(3):186–98. 10.4250/jcvi.2020.0043 32583635 PMC7316554

[42] Kweki AG Aiwuyo HO Umuerri EM Aghwana R Oladimeji OM Iloeje UN. Echocardiographic correlates of left atrial function Index among hypertensive heart failure patients: a cross-sectional study. Cureus. (2023) 15(4):e38013. 10.7759/cureus.38013 37223153 PMC10204615

[43] Zhou Y Zhao CM Shen ZY Zhao X Zhou BY. Mitral early-diastolic inflow peak velocity (E)-to-left atrial strain ratio as a novel index for predicting elevated left ventricular filling pressures in patients with preserved left ventricular ejection fraction. Cardiovasc Ultrasound. (2021) 19:17. 10.1186/s12947-021-00248-z 33894780 PMC8070277

[44] Welles CC Ku IA Kwan DM Whooley MA Schiller NB Turakhia MP. Left atrial function predicts heart failure hospitalization in subjects with preserved ejection fraction and coronary heart disease: longitudinal data from the Heart and Soul Study. J Am Coll Cardiol. (2012) 59(7):673–80. 10.1016/j.jacc.2011.11.012 22322084 PMC3282121

[45] Porpáczy A Nógrádi Á Vértes V Tőkés-Füzesi M Czirják L Komócsi A. Left atrial stiffness is superior to volume and strain parameters in predicting elevated NT-proBNP levels in systemic sclerosis patients. Int J Cardiovasc Imaging. (2019) 35(10):1795–802. 10.1007/s10554-019-01621-w 31093897 PMC6773665

[46] Obokata M Negishi K Kurosawa K Arima H Tateno R Ui G. Incremental diagnostic value of la strain with leg lifts in heart failure with preserved ejection fraction. JACC Cardiovasc Imaging. (2013) 6(7):749–58. 10.1016/j.jcmg.2013.04.006 23747067

[47] Reddy YNV Obokata M Egbe A Yang JH Pislaru S Lin G. Left atrial strain and compliance in the diagnostic evaluation of heart failure with preserved ejection fraction. Eur J Heart Fail. (2019) 21(7):891–900. 10.1002/ejhf.1464 30919562

[48] Fan J-L Su B Zhao X Zhou B-Y Ma C-S Wang H-P. Correlation of left atrial strain with left ventricular end-diastolic pressure in patients with normal left ventricular ejection fraction. Int J Cardiovasc Imaging. (2020) 36(9):1659–66. 10.1007/s10554-020-01869-7 32363448 PMC7438285

[49] Iacoviello M Citarelli G Antoncecchi V Romito R Monitillo F Leone M. Right ventricular longitudinal strain measures independently predict chronic heart failure mortality. Echocardiography (Mount Kisco, N.Y.). (2016) 33(7):992–1000. 10.1111/echo.13199 26864642

[50] Focardi M Cameli M Carbone SF Massoni A De Vito R Lisi M. Traditional and innovative echocardiographic parameters for the analysis of right ventricular performance in comparison with cardiac magnetic resonance. Eur Heart J Cardiovasc Imaging. (2015) 16(1):47–52. 10.1093/ehjci/jeu156 25187607

[51] Motoki H Borowski AG Shrestha K Hu B Kusunose K Troughton RW. Right ventricular global longitudinal strain provides prognostic value incremental to left ventricular ejection fraction in patients with heart failure. J Am Soc Echocardiogr. (2014) 27(7):726–32. 10.1016/j.echo.2014.02.007 24679740

[52] Morris DA Krisper M Nakatani S Köhncke C Otsuji Y Belyavskiy E. Normal range and usefulness of right ventricular systolic strain to detect subtle right ventricular systolic abnormalities in patients with heart failure: a multicentre study. Eur Heart J Cardiovasc Imaging. (2017) 18(2):212–23. 10.1093/ehjci/jew011 26873461

[53] Beyls C Bohbot Y Huette P Booz T Daumin C Abou-Arab O. Usefulness of right ventricular longitudinal shortening fraction to detect right ventricular dysfunction in acute cor pulmonale related to COVID-19. J Cardiothorac Vasc Anesth. (2021) 35(12):3594–603. 10.1053/j.jvca.2021.01.025 33558133 PMC7832272

